# Anterior Cruciate Ligament Tissue Engineering: Biological Principles, Engineered Substitutes, and Preclinical Outcomes

**DOI:** 10.3390/bioengineering13040442

**Published:** 2026-04-10

**Authors:** Franck Simon, Christophe Caneparo, Jadson Moreira-Pereira, Stéphane Chabaud

**Affiliations:** 1 Pathology Division, Christie Innomed, Montréal, QC J7R 0C3, Canada; 2 Department of Pediatrics, Gynecology and Obstetrics, Faculty of Medicine, Geneva University Hospitals, University of Geneva, 1205 Geneva, Switzerland; 3 Biomaterials and Spectroscopy Research Group, School of Arts, Sciences and Humanities, University of São Paulo—USP, São Paulo 03828-000, SP, Brazil; j.pereira@unifesp.br; 4 Centre de Recherche en Organogenèse Expérimentale de l’Université Laval/LOEX, Centre de Recherche du CHU de Québec-Université Laval, Axe Médecine Régénératrice, Québec, QC G1J 1Z4, Canada

**Keywords:** anterior cruciate ligament, tissue engineering, collagen hydrogel

## Abstract

The rising popularity of sports practiced without adequate preparation has increased the incidence of anterior cruciate ligament (ACL) injuries, particularly among young individuals. Because the ACL has a very limited intrinsic healing capacity, surgical reconstruction—most often using autologous grafts—remains the standard of care. However, current techniques frequently lead to donor-site morbidity and do not consistently restore long-term joint stability, contributing to early post-traumatic osteoarthritis in active patients. Over the past decades, tissue engineering (TE) has opened promising avenues for developing biological substitutes capable of overcoming these limitations. Despite substantial progress, no strategy has yet demonstrated reliable and clinically validated functional regeneration of the human ACL. Meanwhile, artificial intelligence is emerging as a complementary tool for diagnosis, surgical planning, biomechanical assessment, and personalized reconstruction strategies. This review aims to provide a comprehensive overview of current TE-based approaches for ACL repair and reconstruction, analyzes their biological and biomechanical limitations, and discusses emerging concepts that may enhance future clinical outcomes. We first summarize the fundamental principles of tissue engineering, then examine the major strategies proposed for ACL regeneration—highlighting their respective strengths and shortcomings—and finally outline perspectives for a novel approach currently under development.

## 1. Introduction

Anterior cruciate ligament (ACL) rupture is one of the most common knee injuries in sports, with an annual incidence of 68.6 per 100,000 persons in the United States of America (USA) [1]. ACL reconstruction is most frequently performed in adolescents and young adults [2,3]. Unlike other ligaments of the knee joint, the ACL does not heal spontaneously once ruptured [4]. Sex-based disparities exist, which can be partially explained by progenitor cells producing less collagen in women [5]. As a key stabilizer of the knee and a critical contributor to proprioception, it has been extensively studied. Although ACL reconstruction using autografts remains the clinical standard, long-term comparative studies show no clearly superior graft choice over more than 10 years, indicating that no universally optimal long-term reconstructive strategy exists [6].

Orthopedic surgeons choose the most appropriate surgical technique based on injury severity and the number of structures affected. Surgical approaches have evolved little over the past three decades. Commonly used grafts include autologous or allogeneic tissues such as hamstring tendons (semitendinosus or gracilis), segments of the autologous quadriceps tendon, or the bone–patellar tendon–bone (BPTB) graft, which is still considered the gold standard [7,8]. Reconstruction requires the insertion and fixation of the graft through tunnels drilled in the lateral femoral condyle and the intercondylar region of the tibia [4]. However, tibial tunnel placement remains imprecise in many cases [9], and suboptimal femoral tunnel positioning is strongly associated with postoperative complications and graft failure [10].

Additional clinical limitations include donor-site morbidity, which can prolong recovery and may compromise return to sport. These limitations have been recognized for decades. Importantly, up to 50% of patients treated with standard reconstruction techniques develop post-traumatic osteoarthritis (OA), a condition that significantly impairs quality of life, including in young athletic individuals [11]. ACL deficiency leads to altered neuromuscular control of the quadriceps and hamstring during dynamic activities, contributing to joint instability—a major risk factor for the development of OA [12]. Taken together, these challenges highlight the need for improved surgical strategies to restore and preserve long-term knee function.

Substantial progress in tissue engineering (TE) has enabled the development of biocompatible ACL substitutes designed to offer novel surgical alternatives shortly after injury while reducing autograft-associated morbidity. Biosynthetic grafts have been investigated; however, many materials have demonstrated long-term limitations [13]. Proposed scaffolds include decellularized ligaments or tendons of autologous, allogeneic, or xenogeneic origin [11,14], biomatrices either seeded or not with autologous or heterologous cells [11], and allogeneic or synthetic fibrous constructs—using silk, collagen-based fibers, PCL braided scaffolds, or other nanostructured biomaterials [15,16,17]. Nevertheless, sterilization and preservation processes may adversely affect scaffold structure and mechanical integrity, thereby impairing graft integration [18]. Supplementing scaffolds with growth factors may help restore a microenvironment conducive to cell proliferation, collagen synthesis, and early ligament-like remodeling in vitro and in vivo [19,20].

For a bioengineered ACL (bACL) graft to integrate successfully into the joint, it must be fully biocompatible and capable of supporting host cell colonization. Host-derived fibroblasts infiltrate the matrix to secrete and reorganize collagen fibers, aligning them along physiologically applied tensile forces to progressively reinforce the scaffold. Restoring the hierarchical ultrastructure—from fibrils to fascicles—is essential for long-term mechanical function, as collagen morphology directly governs load transmission in ligaments [21,22,23].

A particularly promising example is a bACL composed of a multilayered collagen matrix seeded with fibroblasts and anchored by two bone plugs connected by a resorbable surgical suture. When implanted in the caprine knee—an animal model with joint dimensions comparable to those of humans—the graft underwent progressive ligament-like remodeling [24] and achieved up to 40% of native ACL tensile strength by 11 months post-implantation [25]. The animals were allowed unrestricted movement on uneven terrain, confirming that the bACL was sufficiently robust to withstand physiological strain and successfully integrate into the joint environment [21,25,26].

Building on this success, further refinements have been investigated to expand clinical applicability and reduce surgical complexity. The use of endobuttons for graft fixation has been evaluated to develop a second generation of implantable bACLs [27]. In parallel, acellular bACL constructs may represent a simpler and more easily standardized alternative for ACL replacement, provided that their in vivo performance is demonstrated.

Together, these advances highlight the growing potential of tissue-engineered ligament grafts to overcome key limitations of current ACL reconstruction techniques. Further investigation is required to optimize graft design, improve biomechanical performance, and validate these next-generation substitutes for clinical translation.

Most existing reviews on ACL reconstruction focus either on the anatomical and clinical aspects of ligament injury or, conversely, on the biomaterials and engineering strategies designed to address it, but rarely bridge these two dimensions. In contrast, the present review integrates detailed biological, molecular, and biomechanical foundations of the native ACL with a comprehensive assessment of current tissue-engineering approaches. By explicitly linking clinical limitations to the structural and biochemical organization of the ligament, this work aims to clarify how fundamental biology should guide the design of next-generation engineered grafts. This integrative perspective provides a unified framework that is essential for developing therapeutically relevant and physiologically informed strategies for ACL regeneration.

## 2. Material and Methods

The literature search for this review was performed primarily through PubMed, which was selected for its broad coverage, standardized indexing, and relevance to biomedical research. Articles were included when full text was accessible either through open-access sources or via Université Laval’s institutional subscriptions. This approach ensured access to peer-reviewed and traceable studies; however, non-indexed works, preprints, or articles available exclusively through other databases may not have been captured. The authors acknowledge this limitation while emphasizing that the included studies represent the most robust and clinically relevant evidence available on ACL tissue engineering.

Artificial intelligence tools were not used for data analysis, scientific writing, or content generation. Microsoft 365 Copilot (licensed Microsoft 365 Copilot experience embedded in Microsoft 365 applications, November–December 2025 release) was used exclusively for linguistic polishing after the full draft had been written by the authors.

## 3. Anatomy and Biomechanics of Anterior Cruciate Ligament

### 3.1. Anatomy of Anterior Cruciate Ligament

**Figure 1:**
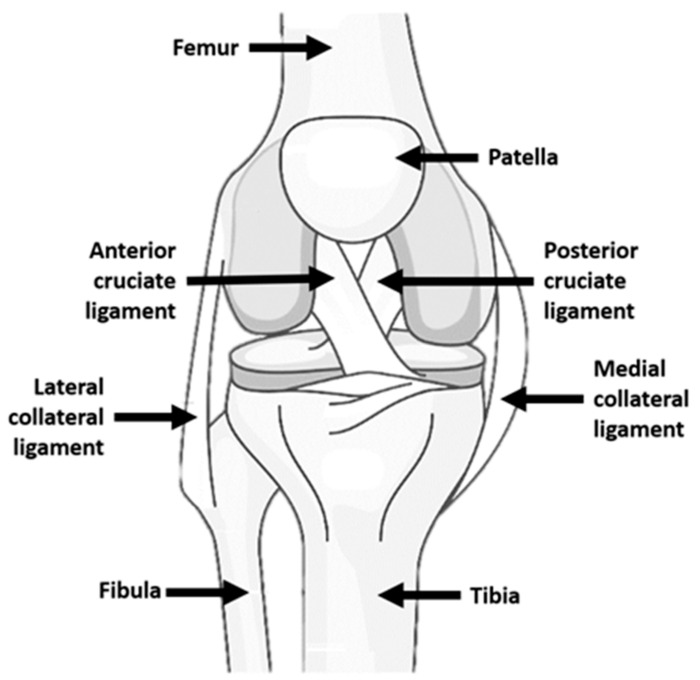
**Knee anatomy.** Anatomy of the human (right) knee showing the main bones and ligaments, including the ACL, PCL, MCL, and LCL. Figure assembled from graphical elements imported in BioRender to be modified and subsequently combined and annotated by the authors. This figure is intended as a simplified conceptual schematic rather than an exhaustive anatomical depiction.

The ACL is one of the primary passive stabilizers of the knee. Situated centrally within the joint, it extends obliquely from the tibial plateau to the medial aspect of the lateral femoral condyle. Through its orientation and mechanical properties, it restricts anterior tibial translation and contributes to the control of axial rotation, particularly when the knee is flexed. Its integrity is therefore essential for maintaining dynamic knee stability during gait, pivoting maneuvers, and rapid directional changes [28,29,30] (Figure 1).

### 3.2. Articular Context

The knee is a synovial joint with a predominantly hinge-like function, enabling flexion–extension coupled with a modest degree of axial rotation. The femoral condyles articulate with the tibial plateau, while the patella tracks within the femoral trochlea [29]. All articular surfaces are covered with hyaline cartilage rich in glycosaminoglycans and hyaluronic acid, providing low-friction movement and resistance to compressive loads.

The joint is enclosed by a fibrous capsule lined internally by a synovial membrane. This membrane produces synovial fluid, which supports cartilage nutrition, modulates lubrication, and maintains a stable biochemical environment. Extra-articular structures, including the medial and lateral collateral ligaments, reinforce mediolateral stability, whereas the menisci act as deformable fibrocartilaginous pads that distribute loads and absorb impact forces. Within this architectural framework, the cruciate ligaments form a functional pair that coordinates anteroposterior and rotational stability of the knee.

### 3.3. Composition and Organization of the Anterior Cruciate Ligament

The ACL is primarily composed of type I collagen, with smaller amounts of type III and additional ECM components such as proteoglycans and glycosaminoglycans [31,32]. These extracellular matrix components are synthesized by resident fibroblasts [33], whose activity depends on the availability of essential metabolic cofactors, such as ascorbic acid [34,35,36]. The hallmark “crimped” or wavy configuration of the collagen fibrils enables the ligament to progressively elongate under load and return to its original shape upon load release [37]. In combination with proteoglycans, elastin, and hyaluronic acid, this fibrillar network provides the ligament with tensile strength, viscoelastic behavior, and multiaxial load-bearing capacity required to sustain the mechanical demands imposed on the knee during daily and athletic activities.

Collagen is the predominant structural component of ligament and tendon extracellular matrices. In mammals, type I collagen accounts for the vast majority of fibrillar collagen, and the ACL is no exception, containing approximately 90% type I collagen along with smaller proportions of type III [38]. Owing to its low antigenicity, high biocompatibility, and ability to attract fibroblasts, purified type I collagen has long been used as a matrix for ligament reconstruction and tissue-engineering applications.

### 3.4. Molecular Architecture

Collagen owes its unique mechanical properties to its triple-helical structure. Each molecule is formed from three α-chains whose amino-acid sequences are enriched in glycine, proline, and hydroxyproline. The repeating Gly-X-Y motif (where X is generally proline and Y hydroxyproline) facilitates tight packing at the center of the helix, while the ring structures of proline and hydroxyproline stabilize chain orientation and limit rotational flexibility. Type I collagen is heterotrimeric (two α1 chains and one α2 chain), whereas type III collagen is a homotrimer composed of three identical α1 chains [39].

### 3.5. Biosynthesis and Post-Translational Modifications

Collagen assembly begins within the rough endoplasmic reticulum, where newly synthesized α-chains undergo extensive post-translational modification. Proline and lysine residues are hydroxylated in reactions requiring oxygen, iron, α-ketoglutarate, and ascorbic acid—cofactors essential for proper triple-helix formation. Deficiencies in these elements impair fibril stability, as illustrated by the clinical features of scurvy [39,40,41].

After hydroxylation and partial glycosylation, the C-terminal propeptides mediate chain recognition and alignment, facilitating formation of the triple helix, which folds from the C-terminal to the N-terminal end. The resulting procollagen molecules transit through the Golgi apparatus and are secreted into the extracellular space, where N- and C-terminal propeptidases cleave the terminal extensions to generate mature tropocollagen [42].

### 3.6. Fibril and Fiber Formation

Tropocollagen molecules spontaneously assemble into staggered arrays, producing the characteristic 67 nm banding pattern observed in fibrillar collagen. Lysyloxidase catalyzes covalent crosslinks between adjacent molecules, stabilizing the initial assemblies into durable microfibrils. These microfibrils aggregate into fibrils ranging from a few tens to hundreds of nanometers in diameter, and fibrils further associate to form collagen fibers measuring 1–10 µm [43,44,45].

Although fibroblasts synthesize the constituent molecules, collagen fibril alignment is primarily guided by external mechanical forces rather than cellular organization alone. Under tension, fibrils orient in the direction of applied stress, ultimately producing the parallel, cable-like architecture typical of ligament tissue.

### 3.7. Hierarchical Organization in the Anterior Cruciate Ligament

In the ACL, collagen fibers are bundled into fascicles—the largest discrete structural units of the ligament. Fascicles typically range from 20 to 400 µm in diameter and contain multiple smaller fiber bundles (1–20 µm) composed of fibrils 25–150 nm in width [46]. This hierarchical arrangement, together with the crimped configuration of the fibrils, allows the ACL to resist tensile, shear, and complex multiaxial loads encountered during physiological movement. The size, orientation, and extent of crosslinking within these structures collectively determine the mechanical performance of the ligament.

### 3.8. Innervation and Proprioceptive Function

The ACL contains a heterogeneous population of mechanoreceptors that underlie its proprioceptive role. Ruffini and Pacinian corpuscles, along with numerous free nerve endings, are located primarily in the subsynovial region [47,48,49,50]. Although they represent only a small fraction of the ligament’s volume [48], these receptors are essential for detecting changes in tension, joint position, acceleration, and intra-articular pressure.

Afferent fibers reach the ligament through branches of the posterior articular nerve [47]. Their guidance depends on adhesive cues within the extracellular matrix, such as laminin and fibronectin, as well as gradients of neurotrophic factors that direct the growth cones of elongating axons [51]. The strong chemoattractive effect of nerve growth factor (NGF) on axonal elongation has been demonstrated in human tissue models, including tooth slice cultures [52].

During graft maturation following ACL reconstruction, similar guidance mechanisms are During graft maturation following ACL reconstruction, similar guidance mechanisms are believed to facilitate neurointegration of the substitute, relying on long-range gradients of attractive and repulsive cues [51]. Sensory feedback from these mechanoreceptors contributes to the reflex modulation of periarticular muscle tone, enabling the neuromuscular system to stabilize the limb in response to ongoing mechanical perturbations [47,53].

### 3.9. Vascular Supply

The ACL receives most of its vascular supply from the middle genicular artery (MGA). This artery branches from the popliteal artery near the proximal femoral condyles, arising just distal to the superior genicular artery and just proximal to the sural artery [29]. The ACL is enveloped by a synovial membrane that carries a delicate vascular network derived from vessels entering the joint both anteriorly and posteriorly. These vessels give rise to a thin periligamentous plexus from which branches penetrate the ligament and extend longitudinally between collagen bundles.

Vascular density is greatest near the tibial and femoral insertions [49], whereas the midsubstance contains relatively few vessels, a feature thought to contribute to its limited intrinsic healing potential [54,55]. During the early stages following implantation of a ligament substitute, synovial fluid can temporarily sustain cellular viability before revascularization progresses from the adjacent synovial membrane. Over time, this ingrowth of vessels contributes to the metabolic support, remodeling, and eventual mechanical maturation of the graft.

## 4. Clinical Issues Associated with Anterior Cruciate Ligament Injury and Current Treatment Strategies

### 4.1. Epidemiology and Mechanisms of Injury

The ACL injuries represent one of the most common traumatic lesions of the knee, particularly in young and physically active individuals. Each year, over 175,000 US patients sustain an ACL rupture [56], most often during noncontact events involving sudden deceleration, pivoting, valgus collapse, or hyperextension forces [57]. Direct contact and collision may also contribute, but in many cases the injury results from a complex interplay of rotational and translational loads applied within fractions of a second. Once disrupted, the ACL rarely regenerates to its native structure or function, distinguishing it from extra-articular ligaments of the knee [58].

### 4.2. Biomechanical and Proprioceptive Roles of the Anterior Cruciate Ligament

The ACL is the principal restraint to anterior tibial translation and a major regulator of internal tibial rotation during load-bearing movement [59]. In the native femur–ACL–tibia complex, ultimate tensile strength exceeds 2000 N [60,61], whereas physiologic joint loading during gait remains well below this threshold. The ligament experiences its highest daily stresses during quadriceps-driven knee extension [62], a factor that directly influences early rehabilitation strategies. Closed-chain exercises are preferred in the acute postoperative period to protect the graft while gradually restoring quadriceps activation and neuromuscular control [63].

### 4.3. Reasons of Anterior Cruciate Ligament Healing Failure

Unlike the medial collateral ligament, the ACL exhibits a strikingly limited intrinsic healing capacity [64]. Although bleeding occurs immediately after injury, the formation of a stabilizing fibrin-platelet scaffold does not take place within the intra-articular environment [65]. The prevailing explanation is the high plasmin activity in synovial fluid, which rapidly degrades fibrin matrices before they can support early cell migration and tissue repair [65,66]. Increased urokinase-type plasminogen activator secretion following joint trauma further enhances fibrinolysis, preventing the establishment of a provisional scaffold that is essential for healing in extra-articular tissues [66]. Additional inhibitory factors include the continuous bathing of the ligament stump in synovial fluid [65,67], post-injury metabolic disturbances [68], and potential intrinsic deficits in the remaining cell populations [64].

### 4.4. Consequences of Anterior Cruciate Ligament Insufficiency: Instability and Osteoarthritis

Loss of ACL function disrupts normal tibiofemoral kinematics, increases stress on secondary stabilizers, and may predispose the joint to early degenerative change [69]. Meniscal injury, altered loading patterns, and episodes of instability all contribute to the development of post-traumatic osteoarthritis [70]. While reconstruction is often recommended for young and active patients, long-term radiographic osteoarthritis appears with similar frequency whether or not the ACL is surgically reconstructed [71]. Nonetheless, reconstruction improves subjective stability, functional performance, and quality of life for patients who wish to maintain higher levels of physical activity [70].

### 4.5. Determinants of Successful Anterior Cruciate Ligament Reconstruction

Surgical outcomes depend on several technical and biological factors. Proper tunnel placement that reproduces the anatomical footprint of the native ligament is critical to restoring physiological tensioning and rotational control. The choice of graft—BPTB, hamstring tendon autografts, or alternative substitutes—affects initial strength, fixation methods, and donor-site morbidity [72,73]. Adequate preconditioning and tensioning of the graft, as well as secure fixation close to the joint line, are essential to provide early stability during rehabilitation. When these principles are applied, medium- and long-term outcomes are generally favorable in terms of knee stability and function [60].

### 4.6. Autografts, Allografts, and Their Limitations

The BPTB autografts have historically been regarded as the biomechanical benchmark for ACL reconstruction, offering strong initial fixation through bone-to-bone healing and mechanical properties approaching those of the native ACL [8,61]. A 10 mm BPTB graft, for example, exhibits stiffness and ultimate load values (210 ± 65 N/mm and 1784 ± 580 N, respectively) that compare favorably with those of the intact femur-ACL-tibia complex (242 ± 28 N/mm and 2160 ± 157 N) [61,74]. This technique also benefits from the presence of bone blocks, which facilitate secure fixation within bone tunnels and contribute to improved knee stability [75].

However, donor-site morbidity remains a major limitation of BPTB autografts. Patients may experience anterior knee pain, loss of motion, extensor mechanism complications, patellar fracture, sensory disturbances, quadriceps weakness, and long-term instability, all of which can compromise postoperative recovery and function [8]. Consequently, many orthopedic surgeons consider it clinically justified to explore alternatives to BPTB grafts.

Hamstring autografts provide an option with reduced donor-site symptoms, yet concerns persist regarding graft diameter, fixation stability, and postoperative laxity. Precise anatomic tunnel placement remains critical for any ACL reconstruction technique, as accurate positioning minimizes graft excursion and notch impingement, thereby optimizing knee stability and range of motion [76,77,78].

Allografts represent another approach, eliminating donor-site morbidity entirely. Cadaveric patellar tendon allografts are mechanically robust and gradually remodeled by host cells after implantation. However, their use is limited by risks of disease transmission, delayed biologic incorporation, immune response, and graft rejection—even in the case of decellularized tissues [65]. Additionally, their higher cost and variable availability can restrict broader clinical use.

Given these limitations, and despite the continued reliance on conventional autografts in clinical practice, there is strong motivation to explore alternative ligament-replacement strategies such as novel biomaterials and tissue-engineered constructs. Advances in tissue engineering—by combining biological and biomechanical expertise—have enabled the development of biocompatible materials and next-generation ACL substitutes that are currently under preclinical and clinical investigation [65,79,80,81,82].

### 4.7. Emerging Reconstructive and Tissue-Engineering Approaches

**Figure 2:**
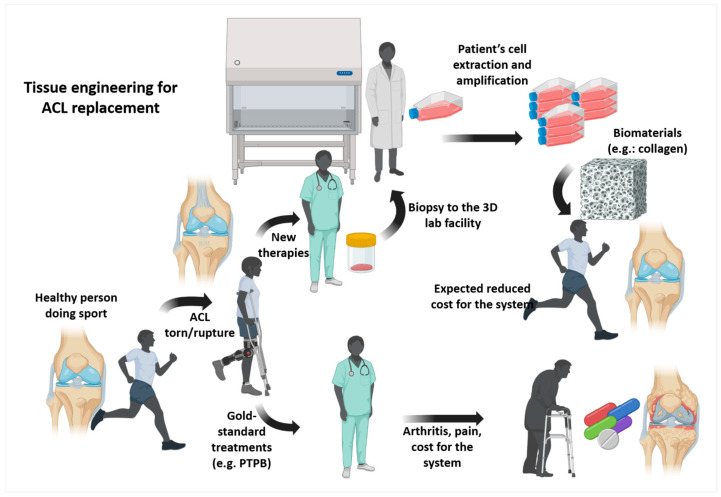
**Tissue engineering workflow for ACL replacement.** Overview of the tissue-engineering process for ACL replacement, from cell extraction and biomaterial preparation to construct fabrication and patient rehabilitation. Figure assembled from graphical elements generated in BioRender and subsequently combined and annotated by the authors.

Advances in tissue engineering and biomaterials science have expanded the possibilities for ACL reconstruction. Approaches under investigation include bioengineered scaffolds designed to support cell infiltration, promote vascularization, and restore progressive mechanical strength; hybrid constructs combining synthetic fibers with biologic matrices; and novel surgical techniques aiming to preserve residual ACL tissue or stimulate intrinsic healing. Regardless of the substitute used, anatomical placement and appropriate mechanical conditioning remain critical determinants of long-term success. As these technologies mature, they may offer viable alternatives that reduce morbidity and improve graft integration compared with traditional autograft or allograft reconstruction (Figure 2).

### 4.8. Reasons for Using Tissue Engineering

Mechanical deficiencies: Native tissues often exhibit structural or functional weaknesses following injury, degeneration, or surgical reconstruction. These mechanical deficits compromise stability and performance, and cannot always be corrected with conventional grafts or repair techniques. Tissue engineering seeks to restore or enhance the biomechanical properties of the damaged structure by generating constructs that more closely reproduce the architecture, composition, and mechanical behavior of the original tissue.

Low regenerative capacity: Many tissues, particularly dense connective tissues such as ligaments, tendons, or cartilage, have a limited intrinsic ability to regenerate due to their low cellularity, limited vascularization, and slow turnover. As a result, spontaneous healing is often incomplete or leads to scar-like tissue of inferior quality. Tissue engineering offers a strategy to overcome these limitations by providing exogenous cells, bioactive cues, and biomaterials that support organized tissue formation and functional restoration.

Unmet clinical need: Current clinical solutions, such as autografts, allografts, or synthetic implants, present significant limitations including donor site morbidity, limited availability, risk of immune reaction or disease transmission, and suboptimal long-term outcomes. Tissue engineering aims to provide safer, more effective, and personalized alternatives that address these shortcomings. By offering the potential for living, patient-specific constructs capable of integration and remodeling, TE responds to a persistent unmet clinical need in orthopedics and regenerative medicine.

## 5. Tissue Engineering

The term “Tissue Engineering” first appeared in a 1993 Science article published by Langer and Vacanti. As with most emerging technologies, TE had been developing in various forms long before it was formally conceptualized; this publication therefore represents the first clear formalization of the concept [83]. TE refers to the in vitro reconstruction of tissues or organs for replacement, repair, or fundamental research. Advances in TE have led to the development of therapeutic solutions for previously unresolved medical problems, such as the lack of efficient small-caliber vascular grafts, rapid and effective wound coverage for burn patients, or engineered tissues for urologenital repair (e.g., [84,85,86,87,88]).

In parallel, TE has enabled the emergence of innovative research models that are particularly valuable at a time when reducing the use of laboratory animals has become imperative [89,90,91]. These models allow research to be conducted in more physiological environments, and crucially using human cells, or even patient-derived cells, paving the way for personalized and precision medicine, particularly in cancer research [92,93,94].

A wide variety of biomaterials have been used in TE [95,96,97]. Earlier synthetic materials include carbon fibers, poly(tetrafluoroethylene) (PTFE), polyester, and polypropylene (PP). More recent options include chitosan, poly(ε-caprolactone) (PCL), poly(ethylene terephthalate) (PET), polyglycolic acid (PGA), as well as polylactic acids (P(LA-CL)/PLA). Natural biomaterials such as collagen, elastin, fibrin, hyaluronic acid, and silk have also been employed. In addition, decellularized organs, used as scaffolds with or without cell seeding, have been explored, as well as hybrid scaffolds combining synthetic and natural materials.

**Figure 3:**
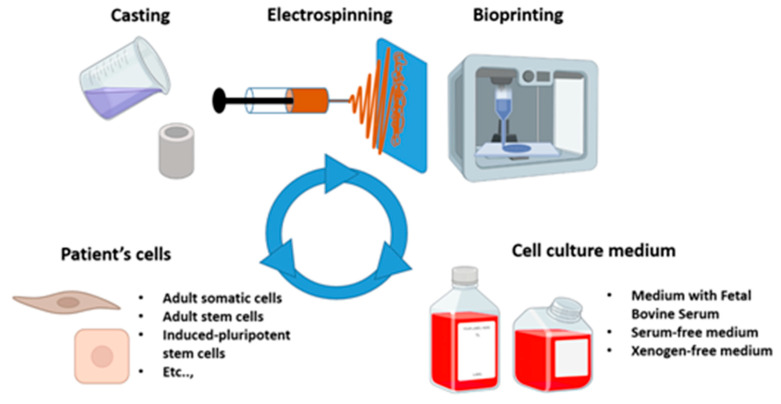
**Main components and fabrication methods in ligament tissue engineering.** Key elements of ligament tissue engineering, including patient-derived cells, culture media, and scaffold fabrication techniques such as casting, electrospinning, and bioprinting. Figure assembled from graphical elements generated in BioRender and subsequently combined and annotated by the authors.

Several techniques have been developed to create scaffolds, such as molding, electrospinning [98,99,100,101], and 3D printing [102,103,104,105,106,107,108,109,110,111] (Figure 3). Other approaches, such as the self-assembly technique, which requires no exogenous biomaterials, have also been developed [112].

Ligament reconstruction provides an excellent example of what these technologies can bring to medicine. The anterior cruciate ligament is simple in composition but physiologically complex due to the mechanical constraints applied to it.

### 5.1. Synthetic Biomaterials

In the following text, bio-resorbable refers to polymers that are completely eliminated from the organism, whereas some biodegradable polymers may leave behind by-products or fragments for extended periods.

The main advantages of synthetic biomaterials are their well-defined composition, relatively low cost, their high tunability, and the possibility of obtaining the desired form directly during fabrication. However, they also present drawbacks: their composition and organization differ from the natural extracellular matrix (ECM), which can affect cell adhesion, proliferation, and differentiation—particularly for fragile cells or those with a high differentiation potential (such as induced pluripotent stem cells) [13].

Nevertheless, significant progress has been made in recent decades regarding the functionalization of synthetic biomaterials, helping to reduce these limitations. Ideally, a biomaterial must withstand physiological mechanical stresses after implantation and be progressively replaced by host tissue, ultimately leading to the formation of a fully autologous and functional neo-tissue. The rate of degradation/resorption is therefore crucial: the biomaterial should disappear at a pace matching its replacement by the host’s newly formed ECM [13].

The first synthetic biomaterials used in the 1970s for ligament TE were carbon fibers, polytetrafluoroethylene (PTFE/Gore-Tex), polyester (Dacron), and PP [72,113,114]. However, clinical outcomes with carbon fibers were disappointing, and their use was discontinued. In the 1980s, PTFE was used; despite excellent mechanical properties, it failed to deliver satisfactory results for ligament applications, mainly due to long-term mechanical fatigue compromising joint stability. Synthetic polyester ligaments, although approved by the Food and Drug Administration (FDA), did not yield better patient outcomes. Some materials, such as polypropylene, were later repurposed to improve the quality of more traditional surgical reconstructions [115].

#### 5.1.1. PCL

PCL’s mechanical and biological properties vary depending on the production technique: molded PCL shows less favorable mechanical characteristics compared to electrospun PCL, though the latter produces a scaffold closer to native tissue [116]. An important advantage of PCL is its ability to be colonized by host cells [117]. PCL degrades in approximately two years, with progressive changes in mechanical properties during degradation [118]. Consequently, PCL is widely used for ligament reconstruction [119,120,121], sometimes in combination with chitosan. PCL–based fibrous scaffolds reinforced with cellulose nanocrystals were engineered using wet-spinning and 3D braiding to closely mimic the structure and mechanical properties of the native ACL, demonstrating excellent biocompatibility and high cell viability. These results underscore the strong potential of PCL as a mechanically robust and clinically relevant scaffold for next-generation ACL repair [16]. Beyond ligament applications, PCL has proven effective in musculoskeletal repair, with PCL-based engineered tendon constructs achieving successful implantation and functional restoration in a porcine model [122].

#### 5.1.2. PGA and PLA

PGA degrades more rapidly than PLA. Both biomaterials and their derivatives release lactate during degradation [123]. Although local lactate release does not necessarily constitute a systemic risk, its effects in sensitive microenvironments could theoretically raise concerns, particularly in oncology [124]. PGA degrades in about two months, progressively losing mechanical strength. This rapid degradation may be too fast to allow proper ECM replacement and host cell colonization. PLA, with a slower degradation rate and persistence of several years after implantation, is likely a suboptimal but more suitable option than PGA for ligament TE. Other well-known synthetic biomaterials, such as poly(lactic-co-glycolic acid) (PLGA) and poly-L-lactic acid (PLLA) [125], are frequently used for fixation systems (e.g., screws) in ligament TE.

The biodegradation rate, mechanical properties, and biological behavior of synthetic biomaterials can be modified using various techniques [126,127]. Copolymerization allows modulation of degradation rates. Material functionalization, for instance by adding proteins or peptides such as Arginine-Glycine-Aspartate (RGD) sequences, can significantly influence cell migration, proliferation, and differentiation. Surface modifications, including plasma treatments, can also be applied to physically or chemically alter material surfaces, for example to adjust hydrophilicity. The use of hybrid scaffolds combining synthetic and natural biomaterials will be described later.

### 5.2. Natural Biomaterials

Natural biomaterials used in tissue engineering are often derived from components of the native ECM. Unlike biosynthetic materials, they inherently provide a microenvironment that is more conducive to cell migration, proliferation, and differentiation when used under appropriate conditions. However, their mechanical properties are generally inferior to those of synthetic scaffolds. As with architectural structures, the simple presence of ECM molecules is insufficient to recreate a functional tissue: mechanical performance arises not only from the biochemical nature of the constituents but, critically, from their hierarchical organization and the interactions among them. This notion is particularly important for ligament engineering, where the native ACL exhibits a highly ordered, anisotropic collagen fiber arrangement essential for tensile strength and load transfer.

#### 5.2.1. Collagen

Representing approximately 30% of total dry body weight, collagens form the most abundant protein family in humans, with 29 known isoforms. They are major components of connective tissues, providing structural integrity and mechanical resistance. Among these proteins, type I collagen is the most highly expressed and the most widely used in tissue engineering, following the pioneering work of Weinberg and Bell [128].

Although collagen hydrogels are popular due to their excellent biocompatibility, their mechanical properties remain weak [27] because they do not replicate the fibrillar alignment and crosslinking characteristic of native ligament ECM. Mechanical conditioning strategies can significantly improve these limitations: controlled fiber alignment and matrix densification can be achieved using bioreactors, cyclic stretching, or flow-induced organization [27,129,130]. These approaches are particularly relevant for ACL reconstruction because they help mimic the native anisotropic architecture required to resist uniaxial tensile loads [131].

#### 5.2.2. Elastin

Elastin is another major ECM component, endowing many connective tissues with elasticity and recoil. Here again, microstructural organization is essential: it is the formation of an interconnected elastin fiber network, rather than the protein itself, that imparts elastic behavior. One of the major challenges in elastin-based biomaterials is therefore the formation of a stable, physiologically relevant network. When artificial crosslinkers are used, they must reproduce the architecture of native elastin to preserve its mechanical properties.

Combining elastin with collagen [132] or with synthetic polymers has been explored to generate composite scaffolds that better recapitulate the viscoelastic profile of ligaments. Such combinations may be particularly advantageous in ACL engineering, where elasticity contributes to energy dissipation during dynamic loading.

#### 5.2.3. Fibrin

During the early stages of wound healing and hemostasis, fibrinogen is cleaved by thrombin to form a fibrin clot, which serves as a provisional matrix supporting cell migration, proliferation, and ECM secretion. Fibrin is also known to promote angiogenesis, a key process for cell survival and tissue repair. However, due to its naturally transient role, fibrin matrices possess very weak mechanical properties and must be rapidly remodeled and replaced by a more durable and organized ECM—an outcome accelerated by the presence of viable cells. Hybrid collagen–fibrin scaffolds have therefore been developed [133], combining the biological activity of fibrin with the improved stability of collagen. For ligament engineering, such hybrids can facilitate early cell infiltration while collagen provides a more resistant backbone for subsequent tissue maturation.

#### 5.2.4. Silk

Among the most innovative natural biomaterials applied in tissue engineering in recent years, silk—one of humanity’s oldest biomaterials—stands out as particularly promising [15,134,135,136,137]. Silk fibers, derived from insect cocoons [138] or spider silk [139], have been used for centuries in textile production, particularly in Asia and Europe. Their mechanical properties, including remarkable tensile strength, elasticity, and durability, make them exceptionally attractive for load-bearing tissue engineering applications.

Silk is biocompatible and exhibits excellent mechanical robustness, with properties that can surpass those of many other natural polymers and even rival certain synthetic materials. For this reason, silk-based scaffolds have gained increasing interest across multiple tissue engineering domains [140,141,142,143], including ligament reconstruction. Their tunability, capacity for functionalization, and slow, controlled degradation profile make them strong candidates for ACL graft development. Additionally, aligned silk fiber constructs can reproduce the anisotropic architecture of native ligaments, making them highly relevant for load-bearing applications.

### 5.3. Hybrid Biomaterials

As mentioned previously, combinations of synthetic and natural biomaterials are increasingly being explored [144,145]. The rationale behind these hybrid constructs is to harness the mechanical strength and tunability of synthetic polymers while preserving the cell-friendly biological features of natural ECM-derived materials. For example, layer-by-layer self-assembled coatings of hyaluronic acid and cationized gelatin have been used to enhance the biocompatibility of poly(ethylene terephthalate) [146]. Other hybrid scaffolds—such as polycaprolactone/collagen/elastin fiber constructs [147] or collagen/polyvinyl alcohol composites [148]—have also been developed for anterior cruciate ligament (ACL) reconstruction. A newly engineered PCL/collagen/hydroxyapatite–nanoclay nanocomposite scaffold has also demonstrated enhanced vascularization, a key requirement for successful ACL graft integration [149]. One approach also combines 3D printing and electrospinning to engineer functionally graded scaffolds mimicking the native ACL structure. It further uses machine-learning-guided design optimization to enhance mechanical performance and produce an optimized scaffold architecture [150]. PCL can also be used with Chitosan to engineer a biocompatible, sterilizable, and functional multilayer braided scaffold for ACL [151]. Although these approaches show promise, further studies are needed to optimize their structural organization, mechanical performance, and long-term integration in vivo.

### 5.4. Decellularization

Among the most promising biomaterials for ligament tissue engineering, decellularized tissues have gained increasing attention owing to recent advances in decellularization protocols [152,153,154]. In principle, no engineered construct can fully replicate the complexity of native extracellular matrix (ECM), making the native tissue itself an ideal starting material. Decellularization aims to remove antigenic cellular components responsible for immune rejection, as well as potential pathogens, while preserving the structural and biochemical framework of the ECM. Once recolonized with the patient’s own cells, the resulting scaffold can approximate the behavior of an autograft and offer an effective solution for ligament reconstruction.

However, several limitations still restrict widespread clinical use. Ethical considerations related to human cadaveric donors remain significant, and stringent regulatory constraints persist. Incomplete or ineffective decellularization carries the risk of residual immunogenicity, inflammatory responses, or graft failure. Such concerns are particularly relevant for ACL allografts, where immune reactions can compromise graft integration and mechanical durability.

Decellularized tissues can be derived from human cadavers or animal sources, and a wide range of chemical, enzymatic, and physical strategies has been developed to remove cellular material. Common approaches include repeated cycles of hypo- and hypertonic solutions combined with detergents such as sodium dodecyl sulfate (SDS), sodium deoxycholate (SD), Triton X-100 or X-200, and milder zwitterionic agents such as CHAPS (3-[(3-cholamidopropyl)dimethylammonio]-1-propanesulfonate). Compared with SDS, CHAPS is less aggressive and better preserves the mechanical integrity of dense connective tissues such as ligaments. These detergents, often used in combination with nucleases (DNases and RNases), enable the disruption of cellular membranes, the solubilization of cytoplasmic proteins, and the degradation of nucleic acids [155].

The preservation of ECM architecture remains the critical challenge. Native ligament function relies on a highly organized collagen type I network, depth-dependent crimp patterns, and specific crosslinking arrangements that determine tensile strength and viscoelastic behavior. Earlier decellularization protocols frequently caused significant ECM disruption, leading to loss of mechanical strength, altered elasticity, reduced collagen fiber alignment, and diminished capacity for host cell differentiation and remodeling. These shortcomings limited the suitability of decellularized scaffolds for load-bearing applications such as ACL reconstruction.

More recent protocols, however, have been designed to minimize structural damage, improve the retention of bioactive cues, and preserve the anisotropic architecture required for ligament functionality [156]. Advances include lower-detergent strategies, enzymatic decellularization with reduced exposure times, perfusion-based approaches, and cryo-processing techniques aimed at maintaining collagen ultrastructure. Despite these developments, further studies are still needed to fully assess the long-term biomechanical performance, recellularization efficiency, and in vivo integration capacity of decellularized ACL scaffolds.

Several recent investigations have evaluated decellularized tissues from diverse sources—including tendon, ligament, pericardium, and small intestinal submucosa—highlighting the potential of these scaffolds for orthopedic applications. Importantly, at least one robust study specifically examining decellularized anterior cruciate ligament grafts has reported that appropriately preserved ECM architecture can support host–cell infiltration, maintain physiologically relevant mechanical properties, and undergo progressive remodeling after implantation. This reinforces the idea that optimized decellularization may offer a viable alternative to traditional allografts or autografts in future ACL reconstruction strategies.

### 5.5. Cells

Tissue engineering combines a biomaterial, dedicated processing techniques and cells selected to match the biological and technical requirements of the protocol. In the context of anterior cruciate ligament (ACL) tissue engineering—when a cellularized construct is targeted—several cell types have been investigated.

The most intuitive choice is the use of autologous cells derived from the injured ligament itself. ACL-derived stem or progenitor cells have shown promising results, notably in enhancing graft–bone integration after reconstruction [157,158] and exhibiting location-dependent differentiation potentials [159].

Other cell sources have also been explored, including umbilical cord-derived mesenchymal stem cells (MSCs), dermal fibroblasts, bone marrow–derived MSCs [121,160], and adipose-derived stem cells (ASCs), the latter improving early biomechanical graft strength in preclinical models [161]. More recently, induced pluripotent stem cells (iPSCs) have drawn interest for their broad differentiation potential [162,163].

Although using cells originating from the target organ is generally preferred for physiological relevance, obtaining sufficient healthy ACL-derived cells can be challenging, particularly when isolation and expansion protocols are not optimized. In such cases, alternative cell types sharing key phenotypic and functional properties—such as dermal fibroblasts—may provide a practical compromise. However, the use of pluripotent cells such as iPSCs requires stringent control of their differentiation to minimize adverse effects, including the risk of tumor formation.

Mitochondria but also extracellular vesicles, including exosomes, from various cells, especially ASCs, have also been used to improve graft take and then outcomes of ligament repair [164,165,166,167].

### 5.6. Mechanical Stimulation

The role of mechanical stimulation to improve mechanical properties, then function, of bioengineered ligament is well known [168,169,170]. Recent advances in ligament and tendon tissue engineering highlight the central role of mechanotransduction in directing collagen architecture and functional maturation [130]. Cyclic strain has been shown to differentially regulate collagen isoform expression, fibril diameter, and alignment, thereby influencing the emerging hierarchical structure of engineered grafts [171]. Load-dependent anabolic responses also vary by sex, suggesting intrinsic biological modulators of ACL mechano-responsiveness [172]. (Contemporary reviews further frame these phenomena within a broader mechanobiology paradigm linking loading regimens to multiscale tissue adaptation [173].

Mechanical stimulation also shapes enthesis maturation, with slow elongation versus rapid cyclic loading driving distinct patterns of fibrocartilaginous development in engineered ACL constructs [174]. At the biochemical level, lysyl-oxidase–mediated crosslinking increases during load-driven collagen maturation, influencing stiffness and hierarchical organization [175]. Mechanosensitive ion channels, particularly PIEZO1, further modulate tendon–ligament stiffness and cellular response to strain, representing a modern entry point into advanced mechanotransduction [176].

Finally, structural evolution—including fibrillogenesis, fibril diameter modulation, and crimp formation—is influenced by scaffold architecture and material cues, with nanostructured or braided biomaterials supporting more ligament-like remodeling [17,177]. These findings collectively outline how mechanical, biochemical, and structural cues synergize to drive graft maturation toward a ligament-like phenotype, informing next-generation ACL tissue-engineering strategies.

## 6. Illustrative Preclinical Example of a Collagen-Based Bioengineered Anterior Cruciate Ligament Substitute

Several tissue-engineering strategies have been explored to overcome the limitations of conventional ACL reconstruction. Among these, collagen-based constructs seeded with fibroblastic cells represent one of the earliest and most extensively characterized approaches for ligament regeneration, and provide a useful illustration of both the potential and the current limitations of this strategy.

In a series of preclinical studies conducted in a caprine model, a bioartificial ACL (bACL) was developed using a type I collagen matrix combined with a temporary load-bearing core and bone or cortical fixation elements. The guiding rationale of this approach was to promote the progressive acquisition of ACL-like structural features by the collagen matrix through host-driven remodeling, rather than relying solely on the initial mechanical strength of the implant [27].

The construct was fabricated in vitro as a multilayered collagen scaffold, initially seeded with fibroblasts or left acellular depending on the experimental condition. Early iterations incorporated bone plugs at both extremities to facilitate anchorage, whereas subsequent designs adopted endobutton-based fixation to improve surgical compatibility and reduce constraints associated with allogeneic bone handling. In all cases, an absorbable suture core was used to assist implantation and provide temporary mechanical support during the early post-implantation phase.

**Figure 4:**
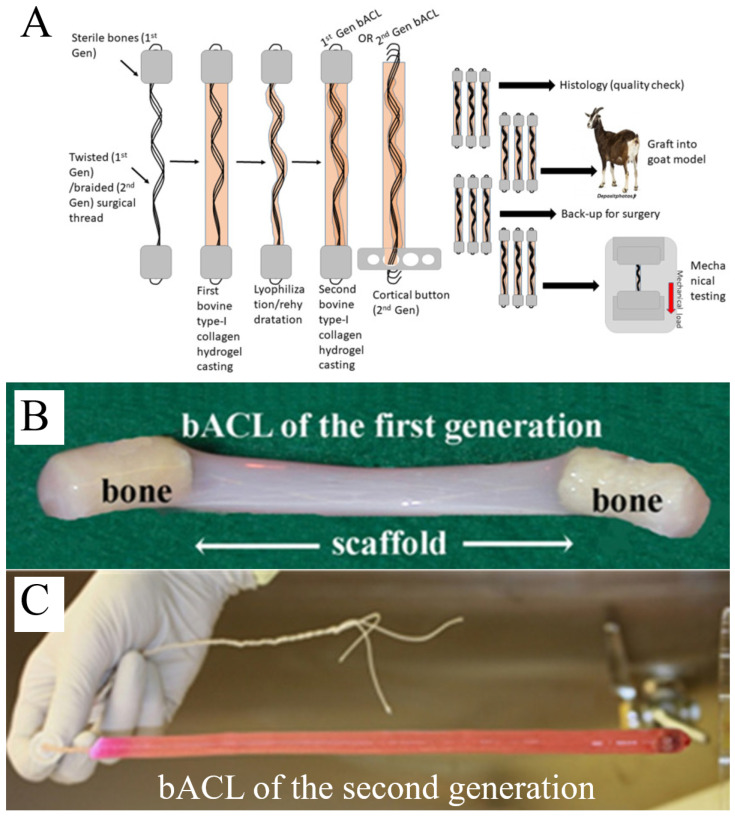
**Representative fabrication strategies and macroscopic features of a collagen-based bioengineered ACL substitute.** (**A**) Schematic overview of a collagen-based tissue-engineering workflow for anterior cruciate ligament replacement, illustrating scaffold formation, incorporation of a temporary load-bearing core, and preparation for implantation. Two illustrative design variants are shown: an early configuration using bone-based anchorage and a later configuration adapted for cortical fixation. (**B**) Macroscopic appearance of a collagen scaffold connecting two anchoring elements prior to implantation. (**C**) Macroscopic view of a collagen-based ligament substitute incorporating a braided absorbable core and a cortical fixation button to facilitate surgical handling and fixation. Images adapted from previously published work [27]. Licensee MDPI, Basel, Switzerland. This article is an open access article distributed under the terms and conditions of the Creative Commons Attribution (CC BY) license (https://creativecommons.org/licenses/by/4.0/).

**Figure 5:**
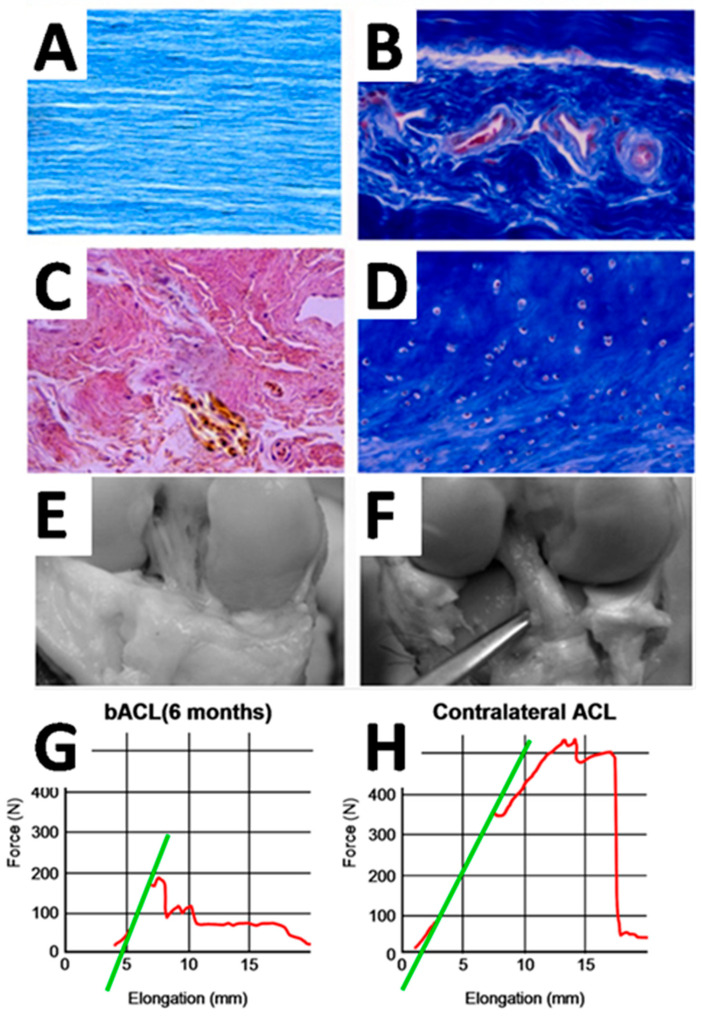
**Histological remodeling and mechanical behavior of a collagen-based bioengineered ACL substitute following mid-term implantation in a large-animal model.** (**A**–**D**) Representative histological sections of an explanted bioengineered ligament substitute at 6 months post-implantation, illustrating collagen organization and cellular remodeling. (**A**) Longitudinal section of the cell-free bACL stained with Masson’s trichrome, showing aligned collagen fibers stained in blue and oriented parallel to the applied tension. (**B**) Masson’s trichrome staining revealing fibrovascular tissue infiltration, with neovascular structures containing red/pink-stained endothelial cells within the remodeled scaffold. (**C**) Histological section showing nerve-like structures embedded within the collagen matrix (brown-stained fibers), consistent with nerve endings. (**D**) Chondrocytes observed at the interface between the ligament scaffold and the bone anchor (Masson’s trichrome staining). (**E**) Gross appearance of the explanted bioengineered construct after 6 months in vivo. (**F**) Gross appearance of the contralateral native ACL shown for qualitative comparison. (**G**) Representative tensile load–elongation curve of the bioengineered construct at 6 months. (**H**) Tensile load–elongation curve of the native ACL tested under identical conditions. In (**G**,**H**), The red curve represents the force–elongation response recorded during tensile testing of the native or bioengineered ACL. The green line corresponds to the tangent drawn over the linear region of the curve and is used to determine the elastic modulus (linear stiffness). Images and mechanical data adapted from previously published work [27]. Licensee MDPI, Basel, Switzerland. This article is an open access article distributed under the terms and conditions of the Creative Commons Attribution (CC BY) license (https://creativecommons.org/licenses/by/4.0/).

The following figures (Figure 4 and Figure 5) illustrate one example of a collagen-based ligament tissue-engineering strategy and should be interpreted as representative of a broader class of biologically driven approaches rather than as a definitive or optimized solution (Table 1).

Following implantation in the goat knee, the bioengineered graft underwent a progressive remodeling process characterized by host cell infiltration, collagen reorganization, vascular ingrowth, and the formation of ligament-like tissue. Histological analyses revealed aligned collagen bundles, fibroblast-like cell populations, and gradual integration at the bone–ligament interface, including the appearance of Sharpey-like fibers. Neurovascular elements were also detected during graft maturation, suggesting partial restoration of key ligament features beyond purely mechanical function.

From a biomechanical perspective, tensile testing showed that the remodeled constructs reached approximately 30–40% of the native ACL strength after 10–12 months in vivo, under conditions of unrestricted animal activity and without specific postoperative mechanical conditioning protocols. While these values remain below native levels, they are consistent with those reported for other biological ligament substitutes in comparable models, and highlight both the promise and the limitations of biologically driven ligament regeneration.

Importantly, these results should be interpreted as proof of concept rather than a near-clinical solution. The relatively long maturation time, incomplete recovery of mechanical properties, and variability inherent to biological remodeling remain significant barriers to translation. Nevertheless, this collagen-based strategy provides valuable insights into several key parameters for ACL tissue engineering, including host-mediated matrix remodeling, fixation-dependent integration, and the trade-off between early mechanical stability and biological receptivity.

As such, this example illustrates how tissue-engineered ligament substitutes can achieve durable in vivo integration in a large animal model, while also underscoring the need for further optimization in scaffold architecture, mechanical conditioning, and translational scalability before clinical application can be realistically envisioned.

## 7. Preclinical Trials Conducted During the Last Two Decades (2005–2025)

Only in vivo implantations of true ligament substitutes (synthetic, hybrid, natural, or decellularized scaffolds) have been considered (Table 2, Table 3 and Table 4). Studies in which the intervention consists solely of cell delivery, MSC injection, addition of extracellular vesicles (EVs/exosomes), or biostimulation without a structural ligament substitute are excluded. Only structural grafts that physically replace the ACL are included here. Because very few studies in the past two decades meet these strict inclusion criteria—namely the implantation of a true, structural ACL substitute with reported in vivo outcomes—the number of references available for comparison in the tables is necessarily limited.

Summary of the tables:

Synthetic/Hybrid Materials: Provide a high initial mechanical structure but show slow maturation. Outcomes indicate functional ligament-like remodeling, though still inferior to native tissue (typically ≤20–40% of native strength in the mid-term).

Non-Decellularized Natural Materials: Natural scaffolds (silk, collagen) combined with MSCs support richer tissue regeneration, although their early mechanical strength at implantation can be weaker.

Decellularized Materials: Decellularized grafts offer excellent integration and ligament-like remodeling, often without the need for prior cell seeding, thanks to the preserved native architecture.

## 8. Artificial Intelligence to Improve ACL Reconstruction

Artificial intelligence (AI) is a rapidly developing field whose applications are expanding across many domains, particularly in medicine [185]. Machine learning (ML) enhances the accuracy of predictive models through algorithms capable of recognizing and learning reproducible patterns in large datasets [186,187]. Unsurprisingly, AI-based tools now support systematic literature searches with improved efficiency and reproducibility in medical research [186,187]. Deep learning (DL) architectures, including artificial neural networks, are increasingly employed in robotics to execute highly precise surgical procedures [188,189]. Another emerging application is the design of microrobots for advanced drug-delivery strategies, enabling targeted penetration of otherwise inaccessible tissues such as solid tumors [190].

Several ML models have demonstrated impressive accuracy in predicting postoperative complications [191]. In orthopedics, force platforms coupled with transducers or sensors are widely used to assess joint loading, ground-reaction forces, balance, and gait, although their reliability is often limited by the low fidelity of many sensors [192]. Recent DL-based advances now offer new possibilities for non-invasive estimation of joint forces and injury risk during sports or occupational activities [192].

In the context of knee injuries, DL may eventually support the automated assess-ment of multiple parameters essential for successful ACL reconstruction and long-term stability, including femoral and tibial tunnel positioning, tunnel length, concomitant meniscal lesions, hip-knee-ankle alignment, and tibial slope [10]. DL-based models may also be applied preoperatively to evaluate knee kinematics as a predictor of post-traumatic osteoarthritis [188]. More broadly, AI-based tools are being developed for fracture diagnosis and classification, risk assessment and outcome prediction, intraoperative navigation, robot-assisted surgery, and even pain-management strategies [193,194,195,196,197,198,199].

More recently, ML-based predictive models have been applied specifically to ACL reconstruction. A 2025 systematic review including more than 125,000 patients showed that ML algorithms designed to predict revision ACLR or secondary knee injury achieve strong discriminatory performance (AUC 0.77–0.997), although calibration remains inconsistent across studies [200]. Such models may help identify high-risk patients preoperatively and refine individualized graft-selection and rehabilitation strategies.

AI has also shown potential to enhance postoperative monitoring and rehabilitation. Telerehabilitation systems incorporating “smart” AI-enabled braces have demonstrated functional outcomes comparable—or in some cases superior—to standard in-clinic rehabilitation programs after ACL reconstruction, especially during periods where hospital access is limited [201].

AI-derived models may also support return-to-play decision-making by integrating metrics such as limb symmetry, neuromuscular control, psychological readiness, and movement-quality indicators [202,203].

Finally, recent work suggests that large language models (LLMs) may become useful clinical decision-support tools.

A 2025 study comparing Gemini and ChatGPT-4 demonstrated that Gemini pro-duced responses with greater clarity, completeness, and clinical alignment when eval-uated against AAOS guidelines for ACL reconstruction [204]. Such tools could ultimately assist surgeons in synthesizing evidence, planning interventions, and improving patient counselling.

**Figure 6:**
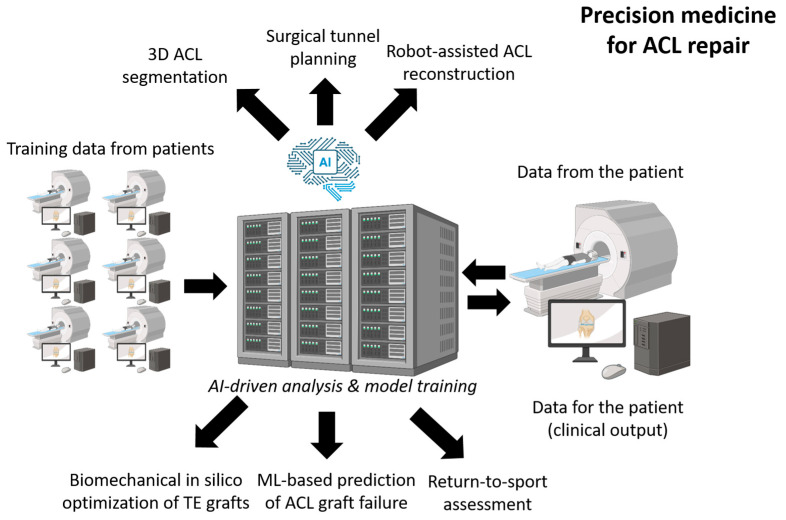
**Artificial-intelligence-enabled precision medicine workflow for ACL reconstruction.** Large multimodal datasets (imaging, biomechanical recordings, clinical variables) collected from patients are used to train AI models for 3D ACL segmentation, surgical tunnel-planning optimization, robot-assisted ACL reconstruction guidance, in silico biomechanical optimization of tissue-engineered grafts, machine-learning–based prediction of ACL graft failure, and objective return-to-sport assessment. These tools collectively support individualized decision-making and may enhance surgical precision, graft longevity, and functional recovery after ACL repair.

Collectively, these advances indicate that AI may improve postoperative knee stability, optimize graft placement, support functional assessment, and assist orthopedic surgeons in addressing the intrinsic limitations of ACL healing and postoperative rehabilitation [200,201,202,203,204,205,206] (Figure 6).

## 9. Conclusions and Perspectives

Anterior cruciate ligament injuries remain a major clinical challenge, particularly in young and physically active individuals. Despite decades of refinement, current reconstruction strategies based on autografts or allografts still have significant limitations, including donor-site morbidity, incomplete restoration of native biomechanics, and a high prevalence of post-traumatic osteoarthritis. These limitations underscore the ongoing need for alternative solutions capable of sustainably restoring knee function.

Over the past two decades, tissue engineering has emerged as a promising strategy to address these shortcomings. Synthetic and hybrid constructs provide strong initial mechanics but show incomplete remodeling. In contrast, natural and decellularized scaffolds promote richer cellular integration at the expense of early structural strength. Preclinical studies consistently demonstrate that no single material or strategy currently fulfills all the biological and biomechanical requirements of a functional ACL substitute.

The main challenge for successful ACL tissue engineering lies in achieving an optimal balance between mechanical competence, biological receptivity, and controlled degradation kinetics. Scaffold architecture, fiber alignment, interface integration, and host-graft interactions remain essential determinants of long-term success. Furthermore, the translation of promising preclinical results to clinical practice remains limited by regulatory constraints, manufacturing complexity, and the need for long-term in vivo validation in clinically relevant large-animal models.

Future progress will likely arise from the convergence of optimized scaffold architectures, mechanical conditioning strategies, advanced decellularization approaches, and better control of host–graft interactions. In parallel, artificial intelligence offers powerful tools for personalized surgical planning, biomechanical prediction, and postoperative monitoring, with the potential to improve both graft placement and long-term joint stability. Together, these innovations pave the way toward next-generation ligament substitutes capable of restoring durable function and reducing the incidence of post-traumatic osteoarthritis.

## Figures and Tables

**Table 1:** Summary of differences between 1st and 2nd generation acellular bACLs (Modified from [27]; Licensee MDPI, Basel, Switzerland). This article is an open access article distributed under the terms and conditions of the Creative Commons Attribution (CC BY) license (https://creativecommons.org/licenses/by/4.0/).

	1st Generation of Acellular bACLs	2nd Generation of Acellular bACLs
Matrix	Bovine Type I collagen hydrogel (can easily be replaced by commercially available recombinant human Type I hydrogels)	idem
Anchorage	Sterile bone plugs	Endobutton (cortical button)
	(limited availability and potential regulatory issues)	
Protection of the graft during implantation	None	Dialysis membrane (efficiency tested by orthopedic surgeons)
Advantages	Surgical procedure similar to standard BPTB procedure	All items used are commercially available and approved by the FDA

**Table 2:** **Synthetic or Hybrid Biomaterials** **(synthetic ± natural).**

Study	Model	Material	Cells	Main Outcomes	Ref.
Cooper 2007	Rabbit	Biomimetic woven PLLA	Cellular + acellular	Good cell infiltration; organized collagen deposition; early ligament-like remodeling; partial load transmission	[178]
Petrigliano 2015	Rat	Electrospun PCL	Mostly acellular	Bone tunnel integration; progressive collagen infiltration; mild immune response; ×3 strength and ×8 stiffness at 12 weeks	[179]
Leong 2015	Athymic rat	Electrospun PCL (protocol)	Potentially cellular	Reliable graft test model; cellular infiltration; lower strength vs. native but confirmed integration	[180]
Lin 2025	Ovine	Electrospun PCL	Potentially cellular	Functional recovery, tissue infiltration throughout its length, joint stability/10 weeks. Graft-bone integration, vascularisation, and early ligament-like remodelling	[181]

**Table 3:** **Natural Biomaterials** **(non-decellularized).**

Study	Model	Material	Cells	Main Outcomes	Ref.
Fan H. 2008	Pig	Silk	Autologous MSCs	Complete ligament regeneration in some animals; Collagen I & III and tenascin-C production; good tissue organization; functional mechanical strength	[182]
Figueroa 2013	Rabbit	Type I collagen	MSCs	33% complete regeneration; organized collagen; peripheral vascularization; superior to cell-free or scaffold-free groups	[183]
Simon F 2021	Goat	Collagen	Cellular + acellular	Functional integrated graft; 18% native strength at 6 months; endogenous migration; histology similar to immature ACL	[27]

**Table 4:** **Decellularized Models (native acellular** **scaffolds).**

Study	Model	Material	Cells	Main Outcomes	Ref.
Edwards 2021	Ovine	Decellularized porcine tendon (pSFT)	Acellular (in vivo repopulation)	Cell recolonization by 12 weeks; complete ligament-like remodeling; Sharpey’s fibers; strength similar to ovine allograft at 26 weeks	[184]
Li 2020	Rabbit	Decellularized allogeneic tendon	Acellular ± reseeding	Greater cell infiltration; improved tendon–bone integration; increased collagen I; moderate immune response	[152]

## Data Availability

No new data were created.
